# Supporting Women Exit Sex Work: A Contribution Analysis of the Exit Doors Here Integrated Care Program in Toronto, Canada

**DOI:** 10.5334/ijic.7700

**Published:** 2024-03-25

**Authors:** Martine Shareck, Pearl Buhariwala, Maha Hassan, Ermelina Balla, Patricia O’Campo

**Affiliations:** 1 Département des sciences de la santécommunautaire, Universitéde Sherbrooke, Sherbrooke, QC, Canada; 2 MAP Centre for Urban Health Solutions, St. Michael’s Hospital, Toronto, ON, Canada; 3 Elizabeth Fry Toronto, Toronto, ON, Canada; 4 Dalla Lana School of Public Health, University of Toronto, Toronto, ON, Canada

**Keywords:** contribution analysis, critical time intervention, evaluation, exiting sex work, marginalized populations, sex work, social determinants of health

## Abstract

**Introduction:**

Exiting sex work is a complex process which can be facilitated by integrated action on health and its social determinants such as housing and employment. Few programs offer such coordinated support, and even fewer have been evaluated. We assessed if and how Exit Doors Here, a program anchored in the Critical Time Intervention (CTI) model, facilitated women’s progress towards their goals, and exit from sex work.

**Description:**

We performed a contribution analysis by combining pre-post questionnaire and administrative data from 55 women enrolled in the program (2018–2021), yearly interviews with program staff and peer mentors, and literature reviews to assess program outcomes and mechanisms as described in the theory of change.

**Discussion:**

We found evidence that the program contributed to participants progressing on their pre-employment, housing, income, and sex work exiting goals. We identified four “key ingredients” facilitating success: trust building, collaborative goal setting, connecting with community supports and weekly drop-in sessions.

**Conclusion:**

This rigorous theory-based evaluation provides much needed evidence on the process and effectiveness of an integrated sex work exiting program. Findings regarding key program ingredients can inform other interventions serving similarly marginalized populations.

## Introduction

### Background

Women engaged in sex work face multiple barriers when trying to exit the trade, including challenges related to addictions, physical and mental health, unhealthy personal relationships, legal matters, housing and economic instability and lack of employment skills [1,2]. Albeit less studied, facilitating factors include community, familial and peer support [2]. To be most effective, sex work exiting programs should address the barriers women face in a personalized, comprehensive and integrated fashion [3]. Despite this, few integrated care interventions exist [4] – programs tend to focus on single barriers such as mental health or legal matters – and few have been evaluated, which limits our understanding of if and how such programs work.

One promising approach to support women exiting sex work lies in Critical Time Intervention (CTI), a time-limited, strengths-based and integrated care model which aims to support people during key transitions in their lives. Developed by Susser *et al*. (1997) to assist individuals in overcoming homelessness [5], CTI has since been applied with other populations, including victims of domestic violence [6] and sex workers [7]. CTI is divided into three phases of three months each with decreased intensity over time. During the program, individuals work one-on-one with an assigned case manager to co-develop treatment plans based on their needs (Phase 1), try out the community supports set up for them (Phase 2), and see their care transferred from their case manager to community supports with whom they can work in the long-term (Phase 3). For more information on CTI, see https://www.criticaltime.org/.

### Problem statement

The effectiveness of CTI has been evaluated in relation to mental health (i.e., moving from institutions to community living) [8] and housing (i.e., transitioning from shelters to independent housing) [9]. In a systematic review of CTI applied to marginalized populations dealing with mental health, homelessness or intimate partner violence, Manuel *et al*. (2023) documented positive impacts on reduced homelessness and increased use of health care services. Results were more equivocal for mental health, substance use, quality of life and social support [10]. To our knowledge only one evaluation study has been conducted with regards CTI and exiting sex work, which documented improvements in mental health and reduced involvement in sex work at the end of the program [7].

To fill these gaps in research and practice, the CTI-inspired Exit Doors Here program was developed to support women exiting sex work in Toronto (Canada), and evaluated by a team of independent researchers. In this paper, we describe key ingredients and potential mechanisms of action which helped women progress towards reaching their pre-employment, housing, and income-related goals; improved their readiness to make changes in their lives and strengthened their social support; and led to a decreased involvement in sex work.

### Description of the care practice

Exit Doors Here was developed and implemented by a not-for-profit organization dedicated to delivering women-centered and gender-transformative services, in response to a rise in demand for services from sex workers. In fact, the Toronto east downtown core area is well known for street prostitution where sex workers provide services on the streets and in the city’s strip clubs, massage parlours and underground brothels. However, the fragmented nature of the social and health care service offer which could help address sex workers’ needs is insufficient and inadequate.

Program objectives were to support women who wished to exit sex work in (1) strengthening their social support system comprised of culturally appropriate community-based service providers, peers and family members, (2) developing and consolidating their self-confidence, skills and employability, and (3) progressing towards reaching the goals they set for themselves. This model was deemed to apply to sex work for several reasons. First, it recognizes that exiting is a staged process, and that distinct cognitive and behavioral adjustments may be needed to support behavioural change among populations facing multiple challenges. Second, it acknowledges that relapses and disengagement can occur at any time, which may hinder the ultimate goal of a successful exit from sex work. Third, this model of care allowed women to focus on addressing barriers to their exiting sex work in an integrated fashion rather than independently from one another.

### Program fidelity

Prior to assessing outcomes we evaluated program fidelity, i.e., whether it had been implemented as planned, after the first year of implementation. Most aspects central to the program, such as close and regular follow-up with clients and focused work on their goals were implemented with moderate to high fidelity. Less well implemented aspects concerned the time-limited nature of CTI. In fact, flexibility was required in terms of phase and total program duration. For various reasons, including that the program staff de-prioritized the strict adherence to the three-month per phase limit to accommodate clients’ transient nature, unique gendered needs, and past trauma [11], a number of clients stayed in the program longer than the prescribed nine months. Negative impacts to clients and their progress were minimal; instead, this flexibility was seen as facilitating goal achievement. We therefore moved on to evaluating program outcomes.

## Methods

### Study design

**Figure 1:**
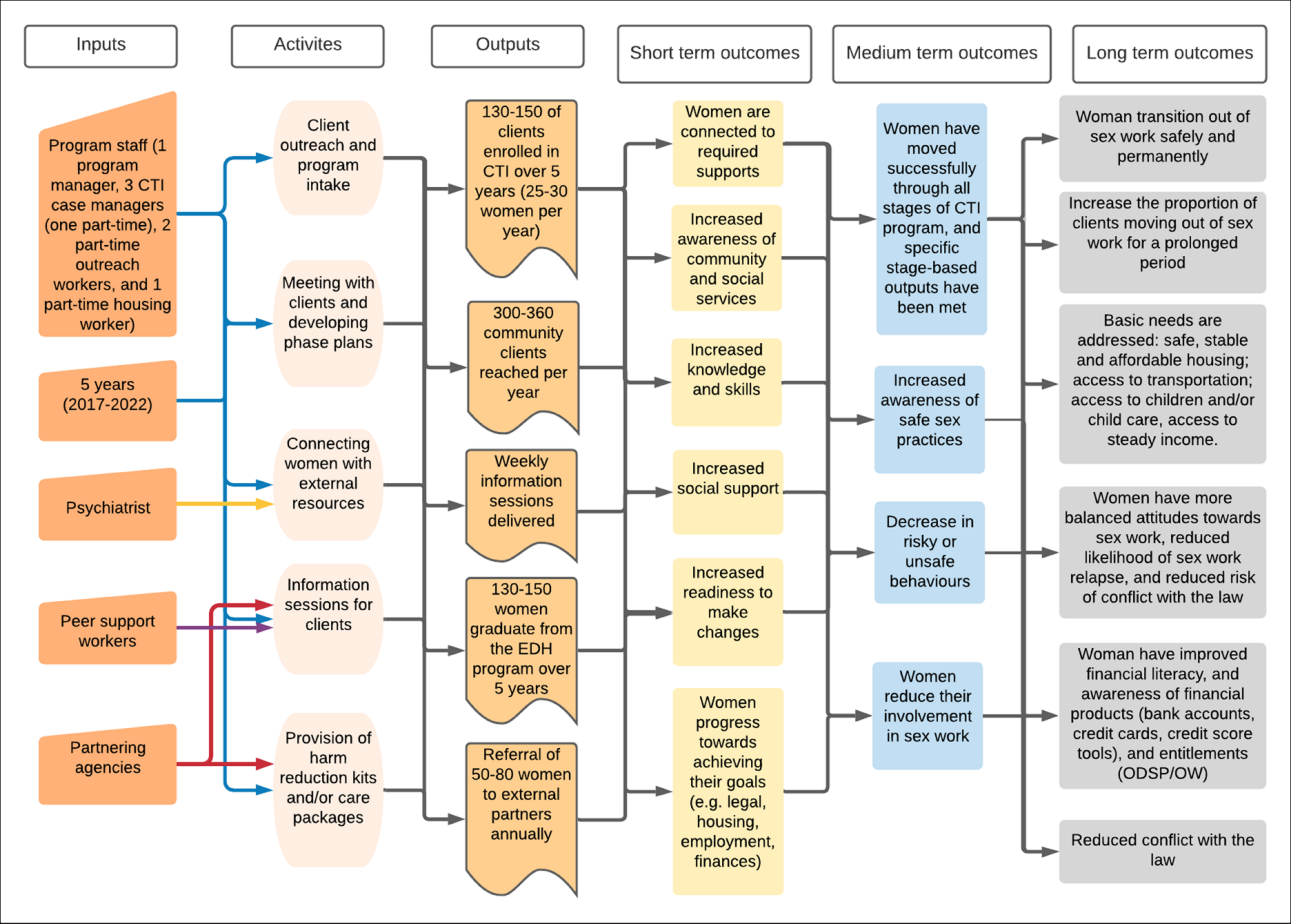
Logic model for the Exit Doors Here program. * We would expect “short term outcomes” to occur over the course of the 9-month program, “medium term outcomes” to occur after the end of the program, and “long term outcomes” to occur years after the program has ended.

A detailed description of the program and evaluation study design, eligibility criteria and procedures are available elsewhere [12]. In brief, we conducted a multi-method process and outcome evaluation, and performed a contribution analysis to assess the contribution which Exit Doors Here made to the outcomes of interest based on the program logic model (Figure 1). Contribution analysis is a theory-based approach particularly well suited to evaluating complex multi-pronged programs which are less suited to experimental designs with control groups [13].

### Participant recruitment

All women enrolled in Exit Doors Here were invited to take part in the evaluation. Case managers provided information to their clients, and evaluators introduced the study to program clients in person at the two program locations and via recruitment pamphlets. Prior to data collection, women, staff and peer mentors provided written or verbal informed consent to participate. Participating women received a 50$ gift card after each questionnaire completion as compensation. The study was approved by the St. Michael’s Research Ethics Board (approval #18–215).

### Data collection

#### Data sources

We combined multiple sources of data. First, participants completed a questionnaire in-person (or virtually during COVID-19) with trained research assistants at baseline (within two weeks of starting Phase 1) and post-intervention (one month after completing Phase 3). Second, participants were asked to grant the evaluators access to the CTI charts in which case managers documented all meetings and progress made. Third, we conducted semi-directed interviews with program staff and peer mentors. Fourth, we accessed the program director’s reports to assess process indicators and fifth, we performed literature reviews.

#### Measures

##### Pre-post questionnaire

We assessed socio-demographics (e.g., age, education, ethnicity) using standard Canadian Census questions. Participants’ scores on the University of Rhode Island Change Assessment (URICA) scale were converted into a readiness to change stage (i.e., pre-contemplation, contemplation, action, and maintenance). Social support was assessed with the 12-item Multidimensional Scale of Perceived Social Support [14], while knowledge of community supports was measured with 10 statements rated on a 5-point Likert scale. We assessed participants’ involvement in sex work after completing the program with the question “How would you describe your level of involvement in sex work this past month?” with response options “not at all”, “rarely” or “frequently”. We used qualitative open-ended questions to explore what participants found helpful from the program, their relationship with their case manager, and factors affecting their involvement in the program including COVID-19 impacts.

##### CTI charts

Participants’ main type(s) of sex work were gathered in their CTI intake form and categorized as “street-based”, “private”, “combination”, and “other” (e.g., trafficked, escorting). To track participants’ progress on pre-employment, housing, and income-related goals, data were extracted from their case and closing notes. Main milestones were defined following discussions with the program team and by identifying activities recurrently reported in the notes. Examples included “connected with external employment supports” (for pre-employment), “housing secured” (for housing), and “applied for social and income assistance” (for income). Each participant was categorized as having achieved each milestone (yes/no).

##### Staff and peer interviews

We conducted semi-directed individual interviews with program staff (seven in year 1, four in year 2 and six in year 3) and peer mentors (two each in years 1 and 2). Interviews identified the barriers and challenges staff and peer mentors faced, parts of the program women engaged with the most/least, areas women needed the most support with, factors influencing their ability to progress through the program, program successes, and COVID-19 impacts.

##### Literature reviews

To gather evidence on CTI and develop the contribution story, we searched selected databases for peer-reviewed articles published from 2000 onwards with the keywords “critical time intervention” or “critical time interventions”, yielding 43 articles, and combinations of keywords related to exiting and sex work, yielding 137 articles.

### Data analysis

To perform the contribution analysis we followed the six steps as defined by Mayne *et al*. (2012): (i) set out the cause-effect issue to be addressed; (ii) develop the postulated theory of change and risks to it; (iii) gather existing evidence on the theory of change, including evidence on assumptions, influencing factors, results and activities; (iv) assemble and assess the contribution story and challenges to it; (v) seek out additional evidence and (vi) review and strengthen the contribution story.

Steps (i) and (ii) were performed collaboratively with the program team through iterative discussions on program objectives, activities, and assumptions which were captured in the logic model (Figure 1). For step (iii) we described socio-demographics and sex work involvement. We compared participants’ baseline and post-intervention questionnaire data to assess pre-post differences in their readiness to change and social support. We computed the proportion of participants who “completely agreed” with each item composing the knowledge of community supports tool, and those who reached each pre-employment, housing, and income-related milestone. We qualitatively summarized women’s answers to open-ended questions and individual staff and peer interview data, and identified supporting quotes. Two experienced research assistants (MH and PB) performed the data analysis, which was regularly discussed with senior researchers MS and PO. We concurrently assessed the strength of the evidence by returning to the literature, triangulating data between sources, and discussing with the program team. Steps (iv) through (vi) were also iterative. Starting from the main program activities, we assembled an initial contribution story tracing activities to outcomes by combining results from previous steps and from literature reviews. To enhance trustworthiness, we discussed the contribution story among evaluators and program staff, and refined and bolstered the theory proposition by seeking additional evidence in the literature or from participant and staff data.

## Results

We describe the sample and summarize how four main program activities, or “key ingredients”, contributed to program outcomes along with risks and challenges to them. We considered the program as a whole, only distinguishing between CTI phases when needed, since the objectives and activities for each phase overlapped. As shown in Figure 1, we could not assume a one-to-one correspondence between activities and outcomes. Hence, activities and processes are not independent from one another; rather, the program as a bundle of activities can be hypothesized as contributing to the observed outcomes.

A total of 155 women were recruited into the program (April 2018–December 2021), 55 of whom enrolled in the evaluation and completed the baseline questionnaire. Of these, 30 also completed the post-intervention questionnaire and 47 granted access to their CTI case notes. Women who dropped out of the program or were lost to follow-up due to mental health, addiction or crises in their lives were excluded from analyses. They did not significantly differ on baseline socio-demographics and sex work involvement from those who were included in the analyses (data not shown).

At baseline, 91% of participants were between 18 and 54 years-old, 86% were born in Canada, 49% identified as Caucasian and 64% had children. Fifty-eight percent had less than a college or trades’ school education, while 9% had some university education. Seventy-six percent were unemployed and 66% had an annual income of 15 000$ or less. A majority of women (89%) were involved in private sex work, street-based sex work or a combination of these (Table 1).

**Table 1:** Baseline socio-demographic characteristics and sex work involvement for 55 women enrolled in the Exit Doors Here evaluation, Toronto, Canada, 2018–2022.


SOCIO-DEMOGRAPHIC CHARACTERISTIC	n (%)

Age group	

18–34 years-old	21 (38)

35–55 years-old	29 (53)

56 years and older	5 (9)

Born in Canada	47 (86)

Ethnic background^a^	

Caucasian	27 (49)

Indigenous	5 (9)

Other	21 (38)

Declined to answer	2 (4)

Highest education level completed	

Grade 10 or below	11 (20)

Grade 11–13	10 (18)

High school	11 (20)

College or trades school	18 (33)

University	5 (9)

Have children, yes	35 (64)

Housing arrangements (n = 47)	

Living in an apartment or house	14 (30)

Living in a shelter	5 (11)

Living in ‘other’ arrangements^b^	14 (30)

Homeless	4 (8)

Missing^c^	10 (21)

Employment status	

Employed	13 (24)

Unemployed	42 (76)

Before-tax income	

$15,000 or less	37 (66)

$15,000–$25,000	11 (20)

$25,000 or more	4 (7)

Declined to answer	3 (7)

**Sex work involvement**	

Involved in sex work	

Yes	52 (95)

No, but at risk of returning	2 (4)

Missing	1 (2)

Type(s) of sex work	

Private	18 (33)

Street-based	9 (16)

Combination	22 (40)

Other (trafficked, escorting, not self-identified)	6 (11)


^a^ “Other” includes Black, South East Asian, Arab, Latin American and mixed backgrounds. ^b^ “Other” includes transitional housing and residential treatment center. ^c^ Eight women did not select “housing” as a focus area and were not asked about housing.

### Contribution story and key ingredients

We focus our description of the contribution story on four “key ingredients” – trust building, collaborative goal setting, connecting women to external community supports, and drop-in sessions – and illustrate how they contributed to selected outcomes and are supported by the literature. The first three are activities built within the CTI model, while drop-in sessions were initiated by the program team.

#### Trust building

A key ingredient for women to engage in the program early on, and remain involved over time, was establishing a trusting relationship with their case manager. A main objective of Phase 1 of CTI, this was nonetheless critical throughout the program. Indeed, building trust in others may be particularly challenging for marginalized groups such as sex workers who disproportionately report past experiences of betrayal from family, friends and service providers [15]. Ttrust has also been identified as central to successful integrated care programs involving marginalized populations as it facilitates clients’ acceptance of case managers or link workers, and a stronger connection to them [16].

In the context of Exit Doors Here, case managers worked to develop a trusting relationship with their clients by employing motivational interviewing techniques [17,18], offering non-judgemental support, adapting to their clients’ needs, and respecting their autonomy in decision-making [15,19]. A recurring theme among women was the importance of their relationship with their case manager. When asked to describe this relationship, one participant commented:

“Amazing, I was so comfortable with her. I consider her a friend, even a mentor. I completely trust her.”

- Evaluation participant, Yr 3 #6

And according to another participant who moved on to become a peer mentor:

“I do know that some of the women actually love their counsellors. This relationship comes from trust and respect.”

- Peer mentor, Yr1 #1

For some participants, gaining trust was a slow process which risked delaying the time-limited intervention, including their being connected with community supports and progressing on their goals. When facing this challenge, case managers remained supportive, in accordance with the host organization’s policy to provide client-centered services which gives women the opportunity to be fully involved in all decisions affecting them, and to do so in their own time. Case managers used active listening and empathy expressed toward their clients’ lived experiences, discussed what made them uneasy or distrusting, and provided more information as needed. Case managers also offered incentives such as toiletries, food vouchers, and public transport or taxi tokens on a case-by-case basis. This type of high-intensity and tailored care model combined with incentives has proven effective in engaging hard-to-reach clients [20], increasing treatment or program compliance [21], strengthening bonds between clients and case managers, and supporting client retention in programs [18]. In Exit Doors Here, participants and peer mentors’ narratives combined to the literature support the hypothesis that strategies used by program staff resulted in clients trusting them and the program.

#### Collaborative goal setting

A second key ingredient of Exit Doors Here was collaborative goal setting via the development of phase plans and completion of empowerment stars. As early as Phase 1 clients identified up to three CTI focus areas they wanted to work on. With their case manager, they then collaboratively assessed their needs and resources relative to these areas and devised a plan to address them. Goals and phase plans were revised and adapted, if needed, at the beginning of Phases 2 and 3. When considering all three phases, the most common focus area was employment/vocational/education (selected by 34 women), followed by housing (n = 31), health and wellness (n = 25), mental health/medication management (n = 21), risky behaviours (n = 12), community and life skills (including income) (n = 10), family and friends (n = 5), parenting skills (n = 4), and children (n = 2). Women also completed empowerment stars with their case manager, which involved reflective conversation and action planning, and helped them assess their progress over time [22].

Although the program was funded to support transition out of sex work and this was an integral part of discussions with participants, this goal was not mandatory. Case managers identified early on the importance of their clients engaging with the program to progress on their goals, and which could, eventually, contribute to their exiting sex work. Supporting this hypothesis is evidence suggesting that women who see the benefits of addressing obstacles and who continuously make progress on clearly defined goals are more likely to exit sex work successfully than those who do not commit to achieving specific goals [23]. Collaborative goal setting may also have contributed to women building trust towards their case managers as it held them accountable [15]. Together, phase plans and empowerment stars helped define a clear direction for women and allowed them to focus on the small steps needed to progress towards successfully exiting sex work:

“The goal setting that we did every few months really helped me steer away from sex work.”

- Evaluation participant, Yr2 #7

“The most valuable thing was doing the empowerment star and having something physical/visual to look at that shows what my priorities were.”

- Evaluation participant, Yr2 #5

Collaborative goal setting and progress assessment were also hypothesized to contribute to increasing women’s readiness to change, which can support program success [24]. However, this was not confirmed here when considering a stage of change framing [25]. Among the 30 participants who completed baseline and follow-up questionnaires, only 7% (n = 2) had moved from “pre-contemplation” or “contemplation” to the “action” stage upon completing the program, while 63% (n = 19) reported no change. This is not entirely surprising given that for most people, behaviour change is not linear, but rather a gradual and staged process which sometimes involves moving backwards. Furthermore, although we assessed readiness to change with a commonly used measures of motivation to change, studies of its predictive validity have found equivocal results [26], so this measure may not have been sensitive or specific enough for our study.

Despite a lack of evidence of progress made in terms of readiness to change as measured here, most participants nevertheless progressed on their goals and reached a number of key milestones over the course of the program with regards pre-employment, housing, and income (Table 2).

**Table 2:** Proportion of participants who reached each key milestone, by CTI focus area.


MILESTONE REACHED	n (%)

**Pre-employment, n = 34**	

Explored employment interests with case manager	16 (47)

Connected with host organization’s employment counsellor	10 (29)

Connected with external employment agencies	15 (44)

Signed up for volunteer positions with program or external agencies	9 (26)

Signed up for entrepreneurial programs	11 (33)

**Housing, n = 31**	

Connected with program housing worker	25 (81)

Connected with external housing worker	17 (55)

Was considered for Special Priority Program housing	22 (71)

Secured housing	18 (58)

Filled housing application, but awaiting decision or accommodation availability	7 (23)

**Income, n = 10**	

Received support with budgeting	6 (60)

Received support filing income tax	5 (50)


All 34 participants who selected pre-employment as a CTI focus area made some progress, including exploring employment interests with their case manager (n = 16), connecting with the host organization’s employment counsellors (n = 10) or external employment supports such as the Canadian Mental Health Association (n = 15), and signing up for entrepreneurial programs (n = 11) or volunteer positions within or outside Exit Doors Here (n = 9) to develop employability skills. By the end of the program, a number of women had taken up employment in education, health care or the film industry, others had returned to school or sought nursing, legal, accounting or computer training, and many were considering training programs directed towards self-employment such as in plumbing. One participant illustrated how the progress they made on their employment and education goals impacted their exit from sex work:

“Since starting the EDH program I have stopped working in the sex trade, I was able to get myself out of it. I now have a unionized job at a local School Board. I just applied at the University to get my bachelor in education to go on to becoming a teacher.”

- Evaluation participant, Yr3 #2

Concerning housing, of the 31 participants who chose this focus area, most sought assistance from the program’s housing worker (n = 25) and/or from an external organization (n = 17), and 22 (71%) were considered for the Special Priority Program housing stream. At the end of the program, 18 participants (58%) had successfully secured housing and seven (23%) were awaiting a decision on their housing application or accommodation availability. Highlighting the interdependency between multiple barriers to exiting sex work and the importance of jointly addressing mental health and housing issues, one participant commented on the collaboration between their case manager and the program psychiatrist:

“The program has been instrumental in making changes. (Case manager) was fabulous in … connecting me to see (program psychiatrist), to get on a priority list for housing specifically for women exiting sex trade. (Program psychiatrist) provided support by writing a letter to support my application and detailed in the letter the importance of housing.”

- Evaluation participant, Yr2 #6

Regarding income-related goals, all participants were offered support with accessing alternative sources of income, and 25 out of 47 for whom data were available applied for financial assistance through the Ontario disability support program, Employment Insurance or the Survivor Support fund. Of the 10 participants who specifically worked on income-related goals, six received help with budgeting (60%) which has been found to support successful exits [20], and five (50%) received help filing their income tax in order to maintain or improve their credit score:

“(Since joining the program) I’ve actually saved some money. I can now budget properly. Without expensive drugs I’m able to save money. I’m on subsidized housing. [Host organization] has helped me with the resources and taught me to be financially responsible. Some of this is taught through the info sessions and some of this we learn through the group of women who attend the program.”

- Evaluation participant, Yr2 #6

In developing the logic model, we determined *a priori* a number of situations which risked delaying women’s progress and goal achievement as planned through collaborative goal setting. First, clients may not have recognized their needs or felt ready to address them, or they may have felt ashamed of needing help. This would have made it difficult to identify the areas they should focus on. To mitigate this, case managers respected their clients’ rhythm and ‘met them where they were’, always remaining flexible as clients’ situations changed through the program. The inability of some women to attend appointments with their case managers and frequent rescheduling also risked delaying progress. To reduce this risk, case managers regularly reminded their clients of upcoming appointments through phone, texts, and emails, ensuring greater appointment compliance [27].

#### Connecting to community supports

Connecting women to community supports is a third key ingredient deemed instrumental to Exit Doors Here. In general, this core CTI activity involves mobilizing resources based on client needs, preferences and service history, monitoring client connections to ensure effective independent functioning of community supports and adapting when necessary, co-developing a long-term care plan with the client, and hosting a transfer-of-care meeting with the client and community supports involved in their longer-term care. This CTI activity is hypothesized to favour overall program effectiveness and sustained impact [28] as well as positive outcomes on the social determinants of health such as employment and income [29].

As part of Exit Doors Here the process of identifying and mobilizing community supports was a collaborative one, drawing on the trust built earlier. Given that a lack of strong partnerships between the program and relevant community supports could have delayed clients’ transfer-of-care, an outreach team used various communication channels such as presentations at external agencies, flyer distribution, and local events to establish new, and strengthen existing, connections. Depending on their needs, women were connected to internal resources including the program’s housing or employment advisors, and to external agencies such as the Centre for Addiction and Mental Health for women experiencing mental health issues. Other agencies provided employment, education and volunteering support through workshops on professional communication, leadership skills, and budgeting. In some cases, these entrepreneurial training programs could lead to funding opportunities for women to launch their own business venture:

“I was involved with the My Startup program, and I successfully graduated. Some of it was over ZOOM (March–June 2020). I learned some new skills such as how to start a new business, which I would like to do eventually.”

- Evaluation participant, Yr2 #4

Within Exit Doors Here, 93% (n = 28) of the 30 participants who completed the follow-up questionnaire reported an increased knowledge of the help available in the community and in how to access these supports, and suggested they had met people they trusted and whom they would turn to for advice if they had trouble. Furthermore, 87% (n = 26) reported benefiting from resources, services, and programs they had not used before, 83% (n = 25) reported their life had improved as a result of knowing where to find community resources and 80% (n = 24) reported the support they received from community resources helped them meet their goals.

There were a number of challenges related to connecting women to community supports, which program staff addressed as they arose. First, clients’ inability to attend scheduled appointments with community supports risked delaying their progress. As suggested by the CTI model, case managers regularly followed up with clients and often accompanied them to their appointments to support greater compliance [8,27]. Second, the inability of community supports to take on client referrals also risked causing delays [29,30]. This was particularly evident during COVID-19 when community organizations were operating at capacity or temporarily closed. To mitigate this, case managers remained in contact with their clients throughout the program and continued making referrals to alternate agencies when needed [29]. Third, since some women may not have been ready to transition from their case managers’ intensive support to the less intensive community supports, they were allowed to continue attending selected program activities such as weekly drop-in sessions. The emotional support offered by case managers and other program clients during this critical time of transition eased women’s transfer-of-care to the community [31] and ultimately contributed to their progressing on their goals and exiting sex work.

#### Weekly drop-in sessions

A fourth activity which stood out as central to the program was the weekly drop-in sessions for current and past clients. These sessions were held throughout the program, even during the COVID-19 pandemic. Workshops were facilitated by case managers or peer mentors on topics such as harm reduction, tenant rights, sexual health, treatment centers, financial management, community programs, and volunteering opportunities. Drop-in sessions provided a safe space where women could meet peers with similar life experiences, express themselves freely [32], and find motivation to stay away from sex work:

“The (drop-in) sessions where people came and spoke about prevention and other topics motivated me to stay away from the field. It made it more comforting because oftentimes women don’t want to exit sex work. It was a safe place for us to talk.”

- Evaluation participant, Yr 3 #3

“There are no fears that anyone is gonna go out and say anything. You’re not gonna be judged, no matter what you say.”

- Evaluation participant, Yr1 #1

They were termed “therapeutic” by one participant, and deemed pivotal in increasing women’s knowledge of external resources, their self-confidence and motivation to change, developing or strengthening their social support network, and gaining employability and life skills that would help them progress towards their goals. Upon graduating from the program, 50% (n = 15) of participants for whom data were available reported an increase in their perceived social support, mostly from family and friends. This may have further contributed to program effectiveness, since an increase in social support has been found to be a mechanism through which CTI might operate [8], although in two studies on the transition from shelter to community housing, one did not find supporting evidence for this [6] and another only found evidence of CTI increasing support from family members [9]. Program graduates also had the opportunity to train as peer mentors who, as part of their role, co-facilitated drop-in sessions. In doing so, they developed self-confidence and essential skills that could help them secure a stable employment outside of the sex trade [24].

COVID-19 and its associated stay-at-home and physical distancing restrictions inevitably posed a risk to the usual conduct of drop-in sessions which were then converted to virtual sessions. Unexpectedly, this change in delivery mode did not negatively affect participation. Although some women preferred in-person sessions, program staff informed us that more women were attending drop-in sessions during the pandemic, including women who did not attend before due to social anxiety or mobility issues. Virtual drop-in sessions thus catered to a more diverse group of women who enjoyed the online format for it saved them time, money and the hassle of commuting [33]. This evidence, along with the robustness of findings despite difficult circumstances such as COVID-19, lends strength to drop-in sessions contributing to women gaining confidence and skills, and progressing towards their goals, in the context of the program.

#### Putting it all together

Overall, the evidence we gathered and triangulated from multiple sources suggests that trust building, collaborative goal setting, connecting to community supports, and weekly drop-in sessions were instrumental in building women’s self-confidence and social support, and in supporting their progress towards their pre-employment, housing and income-related goals. In addition, of the 30 women for whom post-intervention questionnaire data were available, 63 % (n = 19) reported not being involved in sex work and 37 % (n = 11) reported being rarely involved in sex work approximately one month after completing the program. For some women, it was unrealistic to quit sex work entirely within the limited nine months of the program as they needed to remain involved for “*when things get really tight*” *(Evaluation participant, Yr2 #2)*. Nevertheless, as one staff member put it, the integrated nature of the program was key to supporting women’s exit from, or decreased involvement in, sex work:

“… these women have complex needs, they’re extremely marginalized. This program has limited resources; we connect them with housing, financial support and life skills. They need all these to exit sex work safely.”

- Staff, Yr3 #5

## Discussion

### Main findings

In this contribution analysis we demonstrated how key Exit Doors Here program components facilitated participants’ achieving the goals they set for themselves, including exiting sex work. Although participants’ journey through the program were unique, there were similarities. By the end of the program 59% of women with pre-employment goals had signed up for training or skills-improvement activities, 58% of women with housing goals had secured housing, and at least 50% of those with income goals had taken steps to improve their financial situation. All program participants for whom these data were available (n = 30) reduced or ended their involvement in sex work.

We identified four key ingredients which contributed to participants attaining important milestones conceptualized as lying along the process of exiting sex work. Trust building, collaborative goal setting and connecting with community supports are CTI activities, while weekly drop-in sessions were specifically designed for Exit Doors Here. The evidence we presented was strong in that it was anchored in a robust theory of change and resonated with past literature, as well as in light of triangulation of data sources, specificity in recounting, and consistency across samples. Although focused on a population facing multiple systemic challenges, findings from this multi-method evaluation may be relevant to other populations and practices. Indeed the key program ingredients we identified have been documented in other integrated care practices characterized by complex change processes including some targeting youth [34], marginalized communities [16], and people living with chronic conditions [35].

For instance, trust has been found critical in providing integrated care services to marginalized populations [16,19], including sex workers [15,20]. Interestingly, Preble *et al*. (2016) found that sex workers may learn to trust others by modelling trust building practices from their case managers and other program staff [20], which reiterates the importance of building trust early on. This study also provides support for collaborative goal setting as a key ingredient of sex work exiting programs, since it can serve to formalize accountability – from the clients’ perspectives – to progress on their goals. Given that accountability may contribute to trust among this population [20], collaborative goal setting may serve multiple purposes within a program like Exit Doors Here. The third key ingredient we identified was connecting participants to community supports. Akin to patient navigation and linking schemes, it has been associated with improvements in community attitudes towards, and access to, health and social services [16], and increased care completion [36]. Two reviews also found linking and patient navigation schemes to promote patients’ well-being and reduce unmet care needs [29,35]. The value and effects of regular drop-in sessions on skills development and social support have also been documented. For example, “buddy nights” where program participants who would not normally interact were matched and met one-on-one were a valued component of a faith-based sex work exiting program [20].

### Utility of contribution analysis for complex program evaluation

Compared to other evaluation approaches, theory-based contribution analysis is rarely used. Papers tend to focus on the methodological aspects of contribution analyses [37,38] rather than outcome findings [39]. Our experience suggests that contribution analysis is useful and should be considered more often, especially when aiming to understand complex, non-linear change processes in empirical studies with small samples and when having a control group is not possible. We also found contribution analysis helpful to assess the inner workings of a program and how it may bring about changes in clients based on diverse actors’ perspectives. While this increased understanding of program inner workings did not lead us to modify the theory of change, it highlighted how CTI was too rigid an approach for a population such as sex workers, and that flexibility in implementation was required. Our contribution analysis thus helps fill a gap in the literature in general and on exiting sex work, both conceptually and practically.

### Strengths and limitations

Our study has several strengths. We performed a rigorous contribution analysis anchored in a strong theory of change. We triangulated multiple complementary sources of data collected among different groups. Combined to literature reviews, triangulation lent support to the credibility of the claims we made [13,38]. Conducting both a process and outcome evaluation also allowed us to reach a fulsome understanding of the program and its effects. We met regularly with program staff and organized a knowledge mobilization webinar with program clients to discuss preliminary findings, and adjust the contribution story accordingly, thus strengthening plausibility.

Three limitations are worth discussing. First, women who participated in the evaluation and completed the program may not be representative of all female sex workers. Participants who completed both baseline and follow-up questionnaires were not significantly different from those who did not, based on baseline characteristics. However, the program team suggested that women who declined to participate in the first place or who dropped out of the program or were lost to follow-up tended to experience issues which complexified their involvement in such a structured program. Our findings thus likely reflect the experience of women who were more stable and ready to work towards exiting sex work, which should be considered when interpreting results. Second, the URICA scale may not have been as sensitive and specific as required so we were unable to confirm the hypothesis according to which program involvement would increase women’s readiness to change. We nevertheless documented changes in women’s lives, in the form of achievement of important milestones. Third, we were unable to collect data on longer term outcomes, such as whether participants remained in employment and stable housing or sustained their exit from sex work over time.

## Conclusion

Our findings support the idea that sex work exiting programs may benefit from a personalized and integrated approach which takes into account women’s multiple intersecting needs related to health, addiction, housing, employment and training, as documented in “one stop shops” [34]. It also suggests that while exiting sex work may be the ultimate objective of many programs, aiming to change intermediate outcomes and improve the social determinants of health which contribute to women entering and remaining involved in sex work may be equally laudable goals. The key ingredients we documented were common across all three phases of CTI, suggesting that the program as a whole, rather than any single phase or activity, contributed to observed outcomes. While these findings add to the literature on CTI, contribution analysis, and sex work exiting programs, we recommend that further research be conducted to assess the sustainability of CTI-type program effects over time.

## Lessons Learned

- Exiting sex work programs may benefit from an integrated approach targeting both health and its social determinants.
- CTI may be useful to support sex work exiting, but flexibility must be built in to cater to the needs and realities of this particularly marginalized population.
- Contribution analysis can provide a fulsome understanding of complex integrated health and social care programs if it is anchored in a strong theory of change, logic model, and rigorous treatment of multiple complementary data sources.
